# KA-IHO: A Kinematic-Aware Improved Hippo Optimization Algorithm for Collision-Free Mobile Robot Path Planning in Complex Grid Environments

**DOI:** 10.3390/s26082416

**Published:** 2026-04-15

**Authors:** Chunhong Yuan, Yule Cai, Haohua Que, Yuting Pei, Xiang Zhang, Jiayue Xie, Qian Zhang, Lei Mu, Fei Qiao

**Affiliations:** 1 SenseLab, Department of Electronic Engineering, Tsinghua University, Beijing 100084, China; 521031@niuitmo.ru (C.Y.); caiyule0911@outlook.com (Y.C.); haohuaque@outlook.com (H.Q.); peiyuting597@gmail.com (Y.P.); 508513@niuitmo.ru (X.Z.); xjyxjy927927@gmail.com (J.X.); zhangq22@mails.tsinghua.edu.cn (Q.Z.); truemoller@outlook.com (L.M.); 2 Infinity Robotics, Beijing 100083, China; 3 Faculty of Information Measurement and Biotechnical Systems, Saint Petersburg Electrotechnical University “LETI”, St. Petersburg 197022, Russia; 4 Faculty of Control Systems and Robotics, ITMO University, St. Petersburg 197101, Russia

**Keywords:** path planning, swarm intelligence optimization, Hippo Optimization algorithm, kinematic awareness, Lévy flight, Laplacian Ironing Operator, mobile robot, metaheuristic algorithm

## Abstract

Autonomous path planning in obstacle-dense environments remains challenging for swarm intelligence methods due to infeasible initialization, insufficient exploration–exploitation balance, and poor trajectory smoothness for real-robot execution. To address these issues, this paper proposes a Kinematic-Aware Improved Hippo Optimization algorithm (KA-IHO) for mobile robot path planning. The proposed method integrates four components: an elite safety pool initialization strategy to improve feasible solution generation in dense maps, a hierarchical elite-scout update mechanism to better balance global exploration and local exploitation, anti-stagnation mechanisms including a Population Stagnation Restart strategy and a 10-Direction Radial Micro-Search to guarantee high feasibility rates across all map complexities, and a late-stage Laplacian Line-of-Sight Ironing Operator to reduce path redundancy and improve trajectory smoothness. Comparative experiments are conducted on five reproducible grid maps with different complexity levels (40×40 and 80×80), where KA-IHO is evaluated against six representative algorithms, including HO, SBOA, PSO, GWO, ARO, and INFO, over 20 independent runs. The results show that KA-IHO consistently achieves collision-free planning and obtains lower mean fitness values with smaller standard deviations than the compared methods, indicating improved robustness and solution quality. In addition, hardware closed-loop experiments on a differential-drive mobile robot demonstrate that the planned paths can be executed reliably in real environments, with trajectory tracking errors controlled within ±4 cm.

## 1. Introduction

Autonomous navigation and path planning for mobile robots represents one of the most challenging core problems in intelligent robotics, with critical applications in high-value domains including warehouse logistics, disaster rescue, precision manufacturing, autonomous driving, and unmanned aerial vehicle swarm control [1,2]. The essence of path planning lies in finding an optimal trajectory from a start point to a goal point within an obstacle-laden environment, simultaneously satisfying safety constraints, kinematic feasibility constraints, and path optimality constraints. Classical deterministic algorithms such as Dijkstra and A* rely on graph search frameworks and provide completeness guarantees in low-dimensional structured maps; however, their computational complexity grows polynomially or even exponentially with map resolution, making them inadequate for real-time planning in large-scale unstructured environments [3]. Sampling-based methods such as RRT and its variants circumvent the curse of dimensionality by randomly expanding path trees in configuration space, but path quality is significantly affected by sampling randomness, and convergence is extremely slow in high-obstacle-density environments with multiple sharp turns [4].

Over the past two decades, swarm intelligence metaheuristic algorithms—represented by Particle Swarm Optimization (PSO) [5], Ant Colony Optimization (ACO) [6], and Genetic Algorithms (GAs) [7]—have been widely adopted in path planning owing to their simple structure, gradient-free nature, and inherent parallelism. Subsequently, a series of novel swarm intelligence algorithms were proposed and demonstrated competitive performance on path planning benchmarks, including Grey Wolf Optimizer (GWO) [8], Whale Optimization Algorithm (WOA) [9], Harris Hawks Optimization (HHO) [10], Grasshopper Optimisation Algorithm (GOA) [11], and Marine Predators Algorithm (MPA) [12]. Nevertheless, a widely accepted foundational theorem—the No Free Lunch (NFL) Theorem [13]—asserts that no single metaheuristic algorithm can outperform all others across every optimization problem. This theorem has motivated researchers to design increasingly specialized algorithms tailored to specific problem types, and mobile robot path planning, with its distinctive scene-specific characteristics, is precisely such a highly constrained optimization problem.

The Hippopotamus Optimization algorithm (HO) is a recently proposed swarm intelligence algorithm whose design is inspired by the social behavior and spatial ecology of hippos, including their occupation and defense of aquatic territories [14,15], path selection mechanisms during foraging [16,17], and movement patterns under population pressure [18]. HO features a concise structure and few control parameters, conferring good engineering usability. However, in complex path planning tasks, the original HO suffers from three systematic deficiencies: a fragile initialization strategy that drives the initial population directly into collision-heavy regions; a single update mechanism incapable of balancing global exploration and local exploitation; and an absence of geometric post-processing mechanisms, resulting in output trajectories with physical redundancy and physically infeasible sharp turns. Meanwhile, the trajectory executability problem in path planning—namely, whether a planned path can be directly deployed on a real robot’s low-level controller—has been largely overlooked in the existing literature [1,2]. Most existing algorithms optimize path distance alone without considering nonholonomic constraints, rendering their planned results difficult to execute directly on real platforms. This engineering gap constitutes the core motivation of the present study.

Based on the foregoing analysis, existing swarm intelligence path planning methods present the following critical research gaps that have not yet been systematically addressed: (i) in high-obstacle-density environments, random initialization strategies have a high probability of placing the entire initial population in invalid collision regions, forming a “local deadlock” that is difficult to escape; (ii) the exploration–exploitation balance mechanisms of existing algorithms are excessively coarse-grained and unable to simultaneously guarantee optimization accuracy and convergence robustness under the extreme constraints of a small population (N=30) and short iteration budget (T=100); and (iii) generated paths commonly exhibit geometric redundancy such as wall-hugging and sharp-angle turns, and lack a post-processing mechanism coupled with robot kinematic constraints, rendering the planned results directly unusable at the engineering deployment level. To address these challenges, this paper proposes the Kinematic-Aware Improved Hippo Optimization (KA-IHO) algorithm. Here, “kinematic awareness” refers specifically to the property that all output trajectories satisfy the nonholonomic constraints of differential-drive mobile robots—namely, that inter-segment deflection angles remain within executable bounds and no sharp turns are present—so that the planned path can be directly tracked by a low-level controller without additional post-processing. The main contributions of this paper are as follows:

- **Elite Safety Pool Initialization Strategy.** While elite-based initialization has been explored in general metaheuristic design, its systematic application to collision-constrained path planning remains largely unaddressed. The proposed strategy generates a candidate solution pool 100 times larger than the formal population and mandates the selection of zero-collision elite individuals, mathematically eliminating the “initialization deadlock” problem in high-obstacle-density environments and achieving near-100% probability of constructing a safe initial population. The key innovation lies in the path-scene-specific perturbation scheme (Equation (7)) that combines straight-line baseline seeding with adaptive noise scaling, which is specifically designed for grid-map collision landscapes rather than general function optimization.
- **Hierarchical Elite-Scout Iterative Framework.** Although layered population structures and Lévy flight [19,20] and differential mutation [21] are individually well established, their integration into a unified hierarchical framework with a dynamically time-decaying exploration probability tailored to map scale is a novel contribution of this work. The population is ranked by fitness and partitioned into an elite layer (centripetal contraction exploitation) and a scout layer (Lévy flight + differential mutation exploration). Through differentiated update rules, this framework achieves dynamic coordination of global exploration and local refinement, simultaneously ensuring optimization accuracy and convergence robustness under extreme small-population and low-iteration constraints.
- **Late-Stage Laplacian Line-of-Sight Ironing Operator.** Path smoothing post-processors exist in the literature [22,23], but they are typically applied as a separate offline step after optimization terminates. The innovation of the proposed operator is its embedding within the iterative optimization loop with a zero-collision hard constraint, so that geometric straightening and feasibility enforcement are performed jointly and incrementally during the late iteration stage rather than as a decoupled post-process. This greedy geometric refinement mechanism based on midpoint straightening eliminates geometric redundancy and sharp turns, compressing the output path length to its theoretical minimum while ensuring that trajectories satisfy the nonholonomic kinematic constraints of mobile robots and are directly deployable on real platforms [1,2].
- **Anti-Stagnation Mechanisms.** Restart strategies and local search operators are known techniques in the metaheuristic literature; the specific contribution here is their co-design for the grid path planning context: the Population Stagnation Restart (PSR) strategy incorporates an adaptive elite-reduction schedule triggered only within a mid-stage iteration window to avoid disrupting late-stage convergence, while the 10-Direction Radial Micro-Search is purpose-built to resolve pixel-level collision residuals in narrow corridors that standard gradient-free updates cannot address. Collectively, these mechanisms guarantee high success rates across all map complexities.

The remainder of this paper is organized as follows. Section 2 provides a systematic review of related work in the field of path planning, encompassing classical swarm intelligence algorithms, recent state-of-the-art optimization methods, and path post-processing techniques. Section 3 presents the complete design of the KA-IHO algorithm, including problem formulation, initialization strategy, hierarchical iterative framework, dynamic fitness function, anti-stagnation mechanisms, and Laplacian Ironing Operator. Section 4 reports the experimental setup and systematic comparative results, including systematic comparative analysis on five maps of increasing complexity and hardware validation on a real robot platform. Section 5 provides an in-depth discussion of the experimental results. Section 6 concludes the paper and outlines future research directions.

## 2. Related Work

### 2.1. Classical Swarm Intelligence Optimization Algorithms

Swarm intelligence optimization algorithms originate from the mathematical abstraction of collective behaviors observed in nature. Among the most representative foundational algorithms, Particle Swarm Optimization (PSO) [5] and Ant Colony Optimization (ACO) [6] simulate bird flocking foraging and ant pheromone communication behavior, respectively, and have been widely applied in path planning and combinatorial optimization. Genetic Algorithms (GAs) [7], based on natural selection and genetic crossover-mutation mechanisms, approximate global optima through population evolution and constitute an early foundational paradigm for metaheuristic research. The Gravitational Search Algorithm (GSA) [3] introduces Newton’s law of universal gravitation into the optimization framework, providing an early exemplar of physics-metaphor-driven algorithm design. Differential Evolution (DE) [21] achieves outstanding performance in continuous-space optimization through inter-individual difference-vector mutation, and remains an important baseline tool for parametric optimization in path planning to this day [24].

After 2014, a second generation of swarm intelligence algorithms emerged with more sophisticated exploitation–exploration balance mechanisms than PSO, including Grey Wolf Optimizer (GWO) [8], Whale Optimization Algorithm (WOA) [9], Moth-Flame Optimization (MFO) [25], Grasshopper Optimisation Algorithm (GOA) [11], Ant Lion Optimizer (ALO) [26], Sine Cosine Algorithm (SCA) [27], and Salp Swarm Algorithm (SSA) [28]. GWO, in particular, achieves excellent local search capability through its three-tier Alpha–Beta–Delta wolf-pack guidance mechanism and has been widely adopted as a comparative baseline in path planning [8]; WOA implements a concise and effective spiral contraction strategy by simulating the bubble-net hunting behavior of humpback whales [9]. The Multi-Verse Optimizer (MVO) [29] constructs a white-hole–black-hole information transfer mechanism based on cosmological expansion theory; Harris Hawks Optimization (HHO) [10] simulates the cooperative encirclement strategies of raptors; and Marine Predators Algorithm (MPA) [12] achieves efficient global exploration through adaptive switching between Brownian motion and Lévy flight, all of which have been effectively applied to path planning tasks. Nevertheless, these algorithms generally lack dedicated initialization strategies for high-obstacle-density environments and exhibit significant deficiencies in collision penalty modeling precision, frequently generating invalid collision-containing paths in complex constrained environments.

### 2.2. Recent Advances in Metaheuristic Algorithms (2020–2024)

The 2020s have witnessed unprecedented activity in metaheuristic algorithm research, producing a large number of innovative new algorithms. Slime Mould Algorithm (SMA) [30] designs an adaptive weight update mechanism by simulating the foraging and contraction behavior of myxomycetes; Equilibrium Optimizer (EO) [31] constructs concentration update rules based on mass balance equations, achieving a good balance between convergence speed and exploitation accuracy; Aquila Optimizer (AO) [32] simulates four hunting modes of eagles to realize multistage adaptive search; African Vultures Optimization Algorithm (AVOA) [33] and Artificial Gorilla Troops Optimizer (AGTO) [34] simulate the collective behavior of African avians and primates, respectively, demonstrating excellent performance on multimodal function optimization.

Reptile Search Algorithm (RSA) [35], Beluga Whale Optimization (BWO) [36], Artificial Hummingbird Algorithm (AHA) [37], Mountain Gazelle Optimizer (MGO) [38], Honey Badger Algorithm (HBA) [39], and Snake Optimizer (SO) [40] were successively introduced around 2022 and achieved competitive performance on benchmark function tests and engineering optimization problems. Mantis Search Algorithm (MSA) [41] and Nutcracker Optimizer (NO) [42], proposed in 2023, further enriched the bio-inspired algorithm library, while the walrus-behavior-inspired metaheuristic [43], Cheetah Optimizer (CO) [44], and Electric Eel Foraging Optimization (EEFO) [45] represent the latest advances in the field during 2022–2024.

The three baseline algorithms most directly relevant to this paper are the following: Artificial Rabbits Optimization (ARO) [46]—which achieves exploration–exploitation switching through a dual-mode detour foraging and random hiding mechanism and has been among the most frequently cited methods in path planning in recent years; Secretary Bird Optimization Algorithm (SBOA) [47]—which simulates the multiphase hunting and escape behavior of secretary birds and has been applied to obstacle avoidance path planning [2]; and the vector-weighted mean algorithm based on the Runge–Kutta method (INFO) [48]—which designs population update rules drawing on numerical differential equation solving and demonstrates outstanding performance in high-precision continuous-space optimization. Despite their respective merits, all three algorithms suffer to varying degrees from high collision rates, poor robustness, and physically infeasible paths in high-obstacle-density grid maps, which constitutes the core motivation for the improvements proposed in this paper.

### 2.3. Key Techniques in Path Planning: Initialization, Lévy Flight, and Path Smoothing

Initialization strategy is a critically important yet often overlooked component of swarm-intelligence-based path planning. In high-obstacle-density environments, uniform random initialization has a high probability of placing the entire initial population in collision penalty regions, forming an “initialization deadlock” that is difficult to escape. The biological prototype of HO—the common hippopotamus (*Hippopotamus amphibius*)—effectively performs spatial initial positioning through perceptual memory of aquatic territories in nature [14,15,16], exhibiting significant heuristic patterns in habitat utilization and foraging path selection [17,18]. This natural “perception-memory-initialization” mechanism provides the key biological inspiration for the elite safety pool strategy proposed in this paper.

Lévy flight is a class of random walk mechanisms characterized by heavy-tailed step-length distributions following a power-law distribution, capable of producing large-scale jumps with relatively high probability and thereby effectively escaping local optima. Previous studies have demonstrated that embedding Lévy flight into swarm intelligence algorithms can significantly enhance global exploration capability [19] and achieve substantial performance improvements in engineering applications [20]. The difference-vector mutation mechanism in Differential Evolution (DE) [21] has, likewise, been proven to be an effective diversity maintenance technique. In this paper, the combination of Lévy flight and differential mutation as the dual-mode exploration engine of the scout layer constitutes an organic integration and engineering refinement of these two mechanisms.

Path smoothing and post-processing represent the critical step of converting discrete optimized paths into physically executable continuous trajectories. In multiagent cooperative control scenarios, the smoothness of a trajectory and its satisfaction of curvature constraints directly affect the execution performance of low-level controllers [1]; in mobile robot obstacle avoidance tasks, paths containing sharp turns often lead to mechanical wear or even loss of control [2]. Existing path planning evaluation frameworks—such as CEC2014 [49], CEC2022 [50], and the 100-Digit Challenge [51]—are primarily designed for mathematical function optimization and lack dedicated evaluation criteria for path physical executability. Some studies have attempted to introduce curvature penalty terms into the optimization objective [4,52], or improve path quality through hybrid strategies such as PSO-GA [24], but none have fundamentally solved the problem of eliminating late-stage geometric path redundancy. The Laplacian Line-of-Sight Ironing Operator proposed in this paper performs greedy geometric straightening with zero-collision verification as a hard constraint during the late iteration stage, building upon existing work [22,23] to provide a more principled late-stage path refinement mechanism that unifies geometric optimality with kinematic feasibility.

Beyond optimization-based methods, learning-based and control-oriented approaches represent an important complementary paradigm for robot navigation. For instance, Stavrinidis and Zacharia [53] proposed an Adaptive Neuro-Fuzzy Inference System (ANFIS) strategy for autonomous robot collision-free navigation in dynamic environments, demonstrating that learning-based controllers can produce smooth and kinematically executable trajectories in real time. Compared with such data-driven methods, the optimization-based framework of KA-IHO operates without any training phase and does not require prior environment interaction data, making it directly applicable to static complex grid environments where labeled trajectory data are unavailable. Furthermore, whereas ANFIS-based methods typically rely on local sensor feedback for reactive collision avoidance, KA-IHO performs global path optimization over the full map topology, enabling it to plan minimum-length collision-free paths that account for the entire obstacle layout simultaneously. These two paradigms are, thus, complementary: global optimization methods such as KA-IHO provide kinematically feasible reference paths, which can, in turn, serve as reference inputs to local reactive controllers in dynamic deployment scenarios.

## 3. Methods

### 3.1. Problem Formulation

This paper formulates mobile robot path planning as a constrained continuous-space optimization problem. Let the grid map be G of size M×N, where obstacle cells take the value 1 and free-passage cells take the value 0, as defined in Equation (1).

(1) $$G\in {\left\{0,\;1\right\}}^{M\times N},\;G\left(i,j\right)=1\;\left(\mathrm{obstacle}\right),\;G\left(i,j\right)=0\;\left(\mathrm{free}\;\mathrm{cell}\right)$$

Let the start point be $S=(x_{s},y_{s})$ and the goal point be $E=(x_{e},y_{e})$. The path is parameterized by *K* intermediate waypoints, and the decision vector of each agent is defined as in Equation (2). In this paper, K=5, giving a search-space dimensionality of D=2K=10.

(2) $$X=\left(x_{1},\;y_{1},\;x_{2},\;y_{2},\;\ldots ,\;x_{K},\;y_{K}\right)\in R^{2K}$$

The total Euclidean path length $L_{\mathrm{path}}$ is obtained by summing the straight-line distances between consecutive waypoints, as shown in Equation (3), where $p_{k}$ denotes the coordinate pair of the *k*-th segment endpoint in the complete path sequence.

(3) $$L_{\mathrm{path}}\left(X\right)=\sum_{k=0}^{K}{∥p_{k+1}-p_{k}∥}_{2}$$

Collision detection adopts a uniform segment-sampling strategy that samples each path segment at a fixed interval Δs=1/3. The total number of colliding sample points $N_{\mathrm{col}}$ is counted as in Equation (4).

(4) $$N_{\mathrm{col}}\left(X\right)=\sum_{k}\sum_{s}1\;\left[G\;\left(\mathrm{round}\;\left(p_{k}\left(s\right)\right)\right)=1\right]$$

where $p_{k}\left(s\right)=p_{k}+s\cdot \left(p_{k+1}-p_{k}\right)$, $s\in \{0,\;1/n_{s},\;\ldots ,\;1\}$.

The external static fitness function $F_{\mathrm{static}}$ used for final evaluation adopts the pure path distance as the optimization objective, supplemented by a zero-tolerance collision penalty term, as shown in Equation (5). The penalty coefficient $\lambda_{\mathrm{static}}=10^{6}$ ensures that the fitness of any collision-containing path is far higher than that of any safe path.

(5) $$F_{\mathrm{static}}\left(X\right)=L_{\mathrm{path}}\left(X\right)+\lambda_{\mathrm{static}}\cdot N_{\mathrm{col}}\left(X\right),\;\lambda_{\mathrm{static}}=10^{6}$$

In addition to the collision-free constraint, the planned path must satisfy the nonholonomic kinematic constraints of the target differential-drive mobile robot platform. Specifically, the inter-segment deflection angle $\Delta \theta_{k}$ at each waypoint $W_{k}$ must not exceed a prescribed maximum turning angle $\theta_{\max}$, as defined in Equation (6):

(6) $$\Delta \theta_{k}=\arccos\;\left(\frac{v_{k-1}\cdot v_{k}}{∥v_{k-1}∥\;∥v_{k}∥}\right)\leq \theta_{\max},\;k=1,2,\ldots ,K$$

where $v_{k-1}=W_{k}-W_{k-1}$ and $v_{k}=W_{k+1}-W_{k}$ are the direction vectors of consecutive path segments. This constraint captures the physical limitation that a differential-drive robot cannot execute arbitrarily sharp turns at a given forward velocity. Rather than enforcing $\theta_{\max}$ as a hard constraint in the optimization loop—which would dramatically reduce the feasible search space and slow convergence—KA-IHO enforces kinematic feasibility through two complementary soft mechanisms: (i) the smoothness penalty term $C_{\mathrm{smooth}}$ in the dynamic fitness function (Section 3.5), which progressively penalizes large deflection angles throughout the iterative process, and (ii) the late-stage Laplacian Line-of-Sight Ironing Operator (Section 3.7), which geometrically reduces $\Delta \theta_{k}$ at all waypoints via midpoint straightening under a zero-collision hard constraint. Together, these two mechanisms constitute the “kinematic awareness” of KA-IHO: they ensure that the output trajectory is not merely collision-free but also geometrically smooth enough to be tracked directly by the robot’s low-level differential-drive controller without additional post-processing.

### 3.2. Overall Framework of KA-IHO

**Figure 1:**
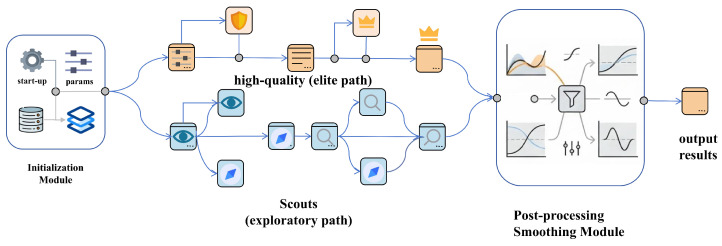
Overall architecture of the KA-IHO algorithm. The left module shows elite safety pool initialization, the middle module shows hierarchical elite-scout iterative optimization with anti-stagnation mechanisms, and the right module shows late-stage Laplacian line-of-sight post-processing for path smoothing.

The KA-IHO algorithm consists of three functional modules connected in series: the oversized elite safety pool initialization module, the hierarchical elite-scout iterative optimization module with integrated anti-stagnation mechanisms, and the late-stage combined Radial Micro-Search and Laplacian Line-of-Sight Ironing post-processing module (see Algorithm 1). As shown in Figure 1, the three modules operate collaboratively through a unified global-best-solution passing mechanism, corresponding respectively to the three core design objectives of “safe initialization”, “efficient processing”, and “optimal output”.

The overall algorithmic flow is presented in Algorithm 1. During the initialization phase, an oversized candidate pool is generated and elite individuals are selected to form the initial population. During the iterative phase, differentiated update rules are executed according to population rank while the global best is maintained. In the late stage, the ironing operator is activated to refine the geometric structure of the path.
**Algorithm** **1** Kinematic-Aware Improved Hippo Optimization (KA-IHO).**Require:** map grid G, start *S*, goal *E*, N=30, *T*, K=5

**Ensure:** best_path, best_fitness, convergence_curve

  1:**Phase 1 – Elite Safety Pool Initialization**  2:Generate pool $X_{\mathrm{pool}}$ ($N_{\mathrm{pool}}=100N$) via Equation (7); evaluate $F_{\mathrm{dyn}}$; sort ascending  3:$X\left[1\ldots N\right]\leftarrow X_{\mathrm{pool}}\left[1\ldots N\right]$; $X_{\mathrm{best}}\leftarrow \arg\min F_{\mathrm{static}}\left(X_{\mathrm{pool}}\right)$  4:**Phase 2 – Hierarchical Iteration with Anti-Stagnation**  5:Init: $C_{\mathrm{stall}}\leftarrow 0$, $f_{\mathrm{prev}}\leftarrow F_{\mathrm{static}}\left(X_{\mathrm{best}}\right)$  6:**for**t=1 **to** *T* **do**  7:   Update $C_{\mathrm{stall}}$; if $F_{\mathrm{static}}\left(X_{\mathrm{best}}\right)<f_{\mathrm{prev}}$: reset $C_{\mathrm{stall}}\leftarrow 0$, $f_{\mathrm{prev}}\leftarrow F_{\mathrm{static}}\left(X_{\mathrm{best}}\right)$  8:   **if** $C_{\mathrm{stall}}\geq 5$ **and** 0.2T<t<0.7T **then**                                                           ▷ PSR  9:         Re-init bottom 70%; $N_{e}\leftarrow \max\left(2,\left\lfloor 0.3N\cdot e^{-C_{\mathrm{stall}}/3}\right\rfloor \right)$; $C_{\mathrm{stall}}\leftarrow 0$  10:    **else**  11:        $\;\;N_{e}\leftarrow \left\lfloor 0.3N\right\rfloor$  12:    **end if**  13:    a←2(1−t/T); update $X_{\mathrm{best}}$ if improved  14:    **for** i=1 **to** $N_{e}$ **do**                     ▷ Elite layer: centripetal contraction, Equation (11)  15:           $X_{\mathrm{new}}\leftarrow X_{\mathrm{best}}-A\cdot \left|C\cdot X_{\mathrm{best}}-X\left[i\right]\right|$; Gaussian perturb (p=0.3)  16:    **end for**  17:    **for** $i=N_{e}+1$ **to** *N* **do**                                       ▷ Scout layer: Equations (16) and (17)  18:       Update via $p_{\mathrm{explore}}\left(t\right)$; boundary reset (Equation (18)); greedy select  19:    **end for**  20:    **Phase 3 – Micro-Search + Laplacian Ironing** (if t>0.65T)  21:    **if** t>0.65·T **then**  22:          **for** k=1 **to** *K* **do**  23:                $W_{k}^{*}\leftarrow \left(W_{k-1}+W_{k+1}\right)/2$  24:                 **if** $F_{\mathrm{static}}\left(X_{\mathrm{cand}}\right)\geq \lambda_{\mathrm{static}}$ **then**                                                   ▷ Collision persists  25:                       Apply 10-dir radial micro-search (Equation (23))  26:                 **else if** $F_{\mathrm{static}}\left(X_{\mathrm{cand}}\right)<F_{\mathrm{static}}\left(X_{\mathrm{best}}\right)$ **then**  27:                       Accept $W_{k}^{*}$; update $X_{\mathrm{best}}$  28:                 **end if**  29:            **end for**  30:      **end if**  31:**end for**  32:**return** best_path, best_fitness, convergence_curve


### 3.3. Oversized Elite Safety Pool Initialization

Traditional swarm intelligence algorithms employ uniform random initialization, which in high-obstacle-density environments can readily cause the entire first-generation population to fall into collision penalty regions, forming an “initialization deadlock”. To address this, KA-IHO constructs a candidate solution pool of size $N_{\mathrm{pool}}=3000$ (100 times the size of the formal population). Each candidate solution is generated by perturbing the straight-line baseline from start to goal with a random noise factor, as in Equation (7), where the noise intensity is adaptively controlled by $\varepsilon_{i}∼U\left(0,1\right)$ to ensure uniform coverage of candidate solutions across the entire map.

(7) $$W_{k}^{\left(i\right)}=S+\frac{k}{K+1}\left(E-S\right)+\varepsilon_{i}\cdot \left(\mathrm{rand}-0.5\right)\cdot L_{\mathrm{map}}$$

where $L_{\mathrm{map}}=\max\left(M,N\right)$ and $\varepsilon_{i}∼U\left(0,1\right)$.

After computing the internal dynamic fitness for all candidate solutions and sorting in ascending order, the top N=30 elite solutions are selected to form the initial population, as in Equation (8). This strategy guarantees, with near-100% probability, that the initial population contains at least one zero-collision individual even in extremely complex maps, fundamentally eliminating the premature deadlock problem.

(8) $$X_{\mathrm{init}}=\left\{X_{\mathrm{pool}}\left[i\right]|i=1,2,\ldots ,N\right\},\;X_{\mathrm{pool}}\;\mathrm{sorted}\;\mathrm{by}\;F_{\mathrm{dyn}}\;\mathrm{ascending}$$

### 3.4. Hierarchical Elite-Scout Iterative Framework

At the beginning of each iteration, the population is sorted in ascending order of $F_{\mathrm{dyn}}$. The top $N_{e}=\left\lfloor 0.3N\right\rfloor$ individuals are assigned to the elite layer and the remaining $N_{s}=N-N_{e}$ individuals to the scout layer, as in Equation (9). The two classes of individuals adopt different update rules, realizing an organic collaboration between local exploitation and global exploration.

(9) $$N_{e}=\left\lfloor 0.3\cdot N\right\rfloor ,\;N_{s}=N-N_{e}$$

#### 3.4.1. Elite Layer Update (Centripetal Contraction)

Elite-layer individuals use the current global best solution $X_{\mathrm{best}}$ as a gravitational center, adopting a GWO-inspired centripetal contraction strategy for high-precision local exploitation. The distance vector D and the updated position $X_{\mathrm{new}}$ are computed as in Equations (10) and (11), respectively.

(10) $$D=\left|C\cdot X_{\mathrm{best}}-X_{i}\right|$$

(11) $$X_{\mathrm{new}}=X_{\mathrm{best}}-A\cdot D$$

The control coefficients *A* and *C* are jointly determined by random vectors $r_{1},r_{2}∼U\left(0,1\right)$ and the time-varying parameter *a*, as in Equations (12)–(14). The parameter *a* decreases linearly from 2 to 0 over the entire iteration cycle, driving the algorithm from early-stage exploration toward late-stage fine-grained exploitation.

(12) $$A=2a\cdot r_{1}-a$$

(13) $$C=2\cdot r_{2}$$

(14) $$a=2\left(1-\frac{t}{T_{\max}}\right)$$

In addition, each elite individual applies a small Gaussian perturbation η∼N(0,1) with probability $p_{\mathrm{pert}}=0.3$, preventing premature collapse of the elite layer to a single local optimum and maintaining the necessary local diversity of the population.

#### 3.4.2. Scout Layer Update (Lévy Flight and Differential Mutation)

Scout-layer individuals are responsible for large-scale global exploration. Their exploration probability $p_{\mathrm{explore}}$ decays dynamically with the iteration progress: cubic decay is applied for large-scale maps (map side length >50) and linear decay for small-scale maps, as in Equation (15).

The exploration probability $p_{\mathrm{explore}}\left(t\right)$ is designed asymmetrically based on two empirical observations. First, large-scale maps (M>50) contain denser obstacle clusters that create deeper local optima, requiring sustained exploration pressure throughout the entire iteration; a cubic decay ${\left(1-t/T_{\max}\right)}^{3}$ maintains high exploration in the early-to-mid stage while accelerating convergence near the end. Second, small-scale maps have fewer feasible corridors, so premature overexploration risks path quality; a linear decay starting from 0.8 rather than 1.0 reflects the fact that the elite safety pool already guarantees a collision-free seed, reducing the need for aggressive early exploration. The initial value $p_{0}=0.8$ and the cubic exponent represent design choices validated empirically on the benchmark maps used in this study.

(15) $$p_{\mathrm{explore}}\left(t\right)=\left\{\begin{array}{cc} 1-{\left(\frac{t}{T_{\max}}\right)}^{3}, & \mathrm{large}\text{-}\mathrm{scale}\;\mathrm{map}\\ 0.8\left(1-\frac{t}{T_{\max}}\right), & \mathrm{small}\text{-}\mathrm{scale}\;\mathrm{map} \end{array}\right.$$

When a scout individual triggers exploration, it switches with equal probability between the Lévy flight mechanism and the differential mutation mechanism. Lévy flight generates a heavy-tailed step length ${\mathrm{step}}_{L}$ with β=1.5 via the Mantegna algorithm, driving individuals to perform large-scale jumps. Its update rule is given in Equation (16).

(16) $$X_{\mathrm{new}}=X_{i}+a\cdot 0.05\cdot {\mathrm{step}}_{L}\cdot \left(X_{\mathrm{best}}-X_{i}\right)$$

where ${\mathrm{step}}_{L}=u/{\left|v\right|}^{1/\beta }$, $u∼N(0,\sigma^{2})$, v∼N(0,1), β=1.5 [19].

The differential mutation mechanism introduces diversity perturbations using the difference vector of two randomly selected, nonrepeating individuals $X_{r_{1}}$ and $X_{r_{2}}$ from the population, enhancing population coverage of the multimodal search space, as in Equation (17).

(17) $$X_{\mathrm{new}}=X_{i}+r\cdot \left(X_{\mathrm{best}}-X_{i}\right)+\frac{a}{2}\cdot \left(X_{r_{1}}-X_{r_{2}}\right)$$

where $r_{1}\neq r_{2}\neq i$ and r∼U(0,1) [21].

When the exploration probability is not triggered, scout individuals perform small-scale Gaussian refinement near the best solution to prevent complete loss of diversity in the late stage. After all update operations, a boundary random-reset strategy (Equation (18)) is applied: each out-of-bounds dimension is reassigned to a randomly sampled position within a narrow band (5% of the domain width) just inside the violated boundary, avoiding the gradient discontinuity of hard clipping and preventing population accumulation at boundary corners.

(18) $$x_{j}=\left\{\begin{array}{cc} lb_{j}+\mathrm{rand}\cdot \left(ub_{j}-lb_{j}\right)\cdot 0.05, & \mathrm{if}\;x_{j}<lb_{j}\\ ub_{j}-\mathrm{rand}\cdot \left(ub_{j}-lb_{j}\right)\cdot 0.05, & \mathrm{if}\;x_{j}>ub_{j} \end{array}\right.$$

### 3.5. Dynamic Fitness Function

The internal iterative phase employs a dynamic fitness function $F_{\mathrm{dyn}}$ that superimposes a time-varying collision penalty and a smoothness cost onto the path length, guiding the population toward broad-area exploration in early iterations and safe-path refinement in later iterations, as in Equation (19).

(19) $$F_{\mathrm{dyn}}=L_{\mathrm{path}}+w_{s}\cdot C_{\mathrm{smooth}}+\lambda_{\mathrm{dyn}}\cdot N_{\mathrm{col}}$$

The smoothness cost $C_{\mathrm{smooth}}$ measures trajectory smoothness by penalizing the cosine of the deflection angle between consecutive path segments, as in Equation (20), where $\theta_{k}$ is the turning angle at the *k*-th waypoint and the weight $w_{s}=2.0$.

(20) $$C_{\mathrm{smooth}}=\sum_{k=2}^{K}\left(1-\cos\theta_{k}\right),\;\cos\theta_{k}=\frac{v_{k-1}\cdot v_{k}}{∥v_{k-1}∥\;\cdot \;∥v_{k}∥}$$

The smoothness penalty term $C_{\mathrm{smooth}}$ serves as the primary kinematic enforcement mechanism during the iterative phase. By penalizing $\left(1-\cos\theta_{k}\right)$, which is a monotonically increasing function of the deflection angle $\Delta \theta_{k}$ defined in Equation (6), the dynamic fitness function continuously steers the population away from sharp-turn configurations. This soft enforcement is intentionally graduated: in early iterations, the low $\lambda_{\mathrm{dyn}}$ allows the population to traverse obstacle regions freely for topological exploration, while the smoothness penalty simultaneously accumulates pressure against kinematically infeasible sharp turns. In late iterations, as $\lambda_{\mathrm{dyn}}$ escalates and the Laplacian Ironing Operator is activated, this pressure converges the population toward trajectories that satisfy the deflection constraint of Equation (6).

The dynamic collision penalty coefficient $\lambda_{\mathrm{dyn}}$ follows a quintic growth schedule with the iteration progress, as in Equation (21). The quintic model starts from a low baseline of 500, permitting the swarm to traverse obstacle regions in early iterations for global topological exploration, and escalates to a peak of $10^{5}$ in late iterations to strictly enforce zero-collision kinematic constraints. At final output, the smoothness term is stripped away, and the pure physical path distance is used for fair performance evaluation.

(21) $$\lambda_{\mathrm{dyn}}=500+99,500\cdot {\left(\frac{t}{T_{\max}}\right)}^{5}$$

### 3.6. Anti-Stagnation Mechanisms: Population Stagnation Restart and Radial Micro-Search

Despite the hierarchical elite-scout framework’s ability to maintain exploration diversity, highly nonconvex environments with U-shaped local minima or pixel-level narrow corridors can still cause the entire population to stagnate. To guarantee full feasibility across all map types, two complementary anti-stagnation mechanisms are introduced.

#### 3.6.1. Population Stagnation Restart (PSR) with Adaptive Elite Reduction

A stagnation counter $C_{\mathrm{stall}}$ tracks the number of consecutive iterations without improvement in $F_{\mathrm{static}}\left(X_{\mathrm{best}}\right)$. When stagnation is detected and the iteration lies within the window $0.2T_{\max}<t<0.7T_{\max}$, the bottom 70% of the population is reinitialized globally using the same elite safety pool perturbation strategy as Phase 1. This window constraint ensures that the restart occurs only during the mid-stage exploration phase, leaving the final 30% of iterations undisturbed for stable convergence. The restart is accompanied by an adaptive reduction in the elite ratio according to Equation (22), which exponentially decays the elite proportion in proportion to the accumulated stagnation depth, forcing a temporary transition into full scout mode.

(22) $$N_{e}=\max\;\left(2,\;\left\lfloor 0.3N\cdot \exp\;\left(-\frac{C_{\mathrm{stall}}}{3}\right)\right\rfloor \right)$$

After the restart, $C_{\mathrm{stall}}$ is reset to zero. The PSR mechanism is triggered at most once per restart window to prevent oscillatory restarts that would undermine convergence stability.

#### 3.6.2. 10-Direction Radial Micro-Search

In extremely narrow corridors where even a single waypoint may remain inside an obstacle after the Laplacian midpoint update, the 10-Direction Radial Micro-Search is activated during the final 35% of iterations (i.e., $t>0.65\cdot T_{\max}$). For each waypoint $W_{k}^{*}$ that still incurs a collision penalty ($F_{\mathrm{static}}\geq \lambda_{\mathrm{static}}$), the operator generates 10 candidate offset positions uniformly distributed in random radial directions at a micro step size δ:

(23) $$W_{k}^{\left(j\right)}=W_{k}^{*}+\delta \cdot \left(\cos\theta_{j},\;\sin\theta_{j}\right),\;\theta_{j}=\frac{2\pi j}{10}+\mathrm{rand},\;j=0,\ldots ,9$$

The candidate with the steepest penalty reduction—i.e., the minimum $F_{\mathrm{static}}$ among the 10 offsets—is accepted to replace $W_{k}^{*}$. This local brute-force search effectively slides collision-trapped waypoints into the nearest feasible corridor gap, eliminating micro-collisions that standard gradient-free updates cannot resolve.

### 3.7. Late-Stage Laplacian Line-of-Sight Ironing Operator

When the iteration reaches $t>0.65\cdot T_{\max}$, the late-stage Laplacian Line-of-Sight (LoS) Ironing Operator is activated jointly with the Radial Micro-Search described in Section 3.6. This operator performs a pointwise straightening operation on each intermediate waypoint $W_{k}$ of the current global best path: $W_{k}$ is replaced by the midpoint coordinate $W_{k}^{*}$ of its two adjacent waypoints, as in Equation (24). This operation is performed for 3 rounds, repeatedly ironing all waypoints in the path.

(24) $$W_{k}^{*}=\frac{W_{k-1}+W_{k+1}}{2},\;k=1,2,\ldots ,K$$

The candidate straightened position $W_{k}^{*}$ is accepted only when the condition $F_{\mathrm{static}}\left(X_{\mathrm{cand}}\right)<F_{\mathrm{static}}\left(X_{\mathrm{best}}\right)$ is satisfied, meaning that the straightening operation must simultaneously satisfy both “shorter path” and “zero-collision constraint not violated.” This mechanism manifests in the convergence curve as a characteristic staircase drop once activated at $t>0.65\cdot T_{\max}$, which is the direct visual evidence of the Laplacian operator eliminating geometric redundancy in one pass.

From a kinematic feasibility perspective, the midpoint straightening operation of Equation (24) has a direct geometric interpretation in terms of the deflection angle constraint defined in Equation (6). Replacing $W_{k}$ with the midpoint $W_{k}^{*}=\left(W_{k-1}+W_{k+1}\right)/2$ moves $W_{k}$ toward the chord connecting its two neighbors, which geometrically reduces the deflection angle $\Delta \theta_{k}$ at that waypoint. Over multiple ironing rounds, this iterative midpoint displacement progressively drives all inter-segment angles toward zero, converging the piecewise-linear path toward a smoother curve with smaller maximum curvature. The combined effect of the smoothness penalty term (Section 3.5) and the ironing operator is, therefore, to enforce, in a soft but systematic manner, the kinematic feasibility condition of Equation (6): the smoothness penalty prevents the population from generating excessively sharp-turn candidates during optimization, while the ironing operator deterministically reduces residual deflection angles in the late stage under the hard constraint of zero collision. This two-stage kinematic enforcement strategy distinguishes KA-IHO from prior path planning methods that either ignore kinematic constraints entirely or apply smoothing only as an offline post-processing step decoupled from the optimization loop [22,23].

## 4. Experimental Results

### 4.1. Experimental Setup

**Figure 2:**
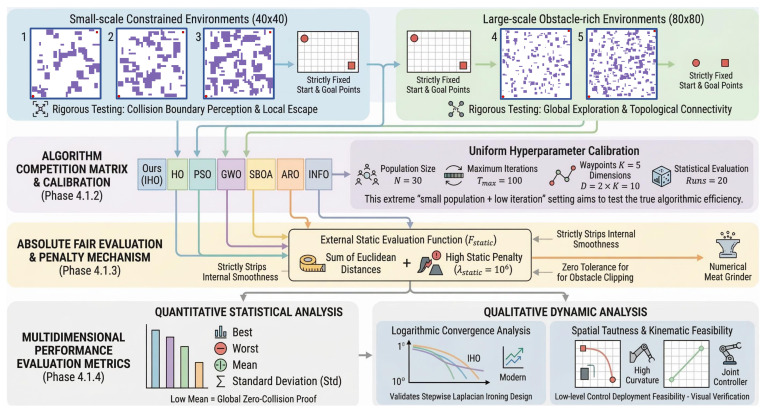
Schematic diagram of the multidimensional benchmark evaluation framework. The framework comprises five test maps, seven algorithms in total (KA-IHO and six baseline algorithms: HO, SBOA, PSO, GWO, ARO, INFO), unified hyperparameter configuration, and a multidimensional performance evaluation metric system.

To systematically evaluate the comprehensive performance of KA-IHO, this section constructs a multidimensional benchmark evaluation framework (Figure 2). The framework encompasses five randomly generated grid maps of increasing complexity: Maps 1–3 are small-scale 40×40 high-obstacle-density environments focusing on testing the algorithm’s collision boundary perception and local escape capability; Maps 4–5 are large-scale 80×80 maze environments that rigorously assess the algorithm’s global exploration ability and topological connectivity recognition. Six baseline algorithms are included: HO, SBOA [47], PSO [5], GWO [8], ARO [46], and INFO [48]. To ensure evaluation fairness, all algorithms are uniformly configured with population size N=30, maximum iterations $T_{\max}=100$, number of waypoints K=5 (dimensionality D=10), and run independently 20 times to ensure statistical reliability.

The five benchmark maps used in this study are generated by a seeded random obstacle placement procedure, ensuring full reproducibility across all experimental runs. Each map is populated with randomly scattered rectangular obstacle blocks of varying size (1×1 to 4×4 cells). A safe zone is enforced around both the start point and the goal point to guarantee path feasibility. The start point is fixed at (2,2) and the goal point at (M−2,N−2) for all maps. Detailed configuration parameters for each map are summarized in Table 1. Statistical performance is reported using five metrics per algorithm per map: success rate (SR, %), valid best, valid mean, valid worst, and valid std. SR measures the fraction of collision-free runs out of 20 independent trials. Valid metrics are computed exclusively from collision-free runs; entries marked “—” indicate zero successful runs (SR = 0%). It is acknowledged that valid mean, being computed only from successful runs, may, in principle, introduce selection bias. However, in the context of this study, such bias is effectively bounded by the collision penalty structure: because $\lambda_{\mathrm{static}}=10^{6}$, any failed run incurs a fitness value on the order of $10^{6}$ or higher, which is three to four orders of magnitude larger than the path lengths of successful runs (typically 55–170 grid units across all maps). Consequently, any algorithm with SR < 100% would exhibit an expected cost over all runs that is overwhelmingly dominated by collision penalty terms, placing it far above any SR = 100% algorithm regardless of its valid mean. This relationship is already captured implicitly by the SR column: an SR of 100% is a necessary condition for an algorithm to be competitive in terms of overall expected cost, and valid mean provides the solution quality comparison among algorithms that satisfy this condition. Therefore, SR and valid mean should be interpreted jointly rather than in isolation—SR establishes the feasibility baseline, and valid mean quantifies path quality among feasible algorithms. This reporting convention follows statistical practices in high-obstacle-density path planning benchmarks [2]. The complete statistical results are presented in Table 2.

### 4.2. Simulation Results in Small-Scale Environments (Maps 1–3, 40 × 40)

**Figure 3:**
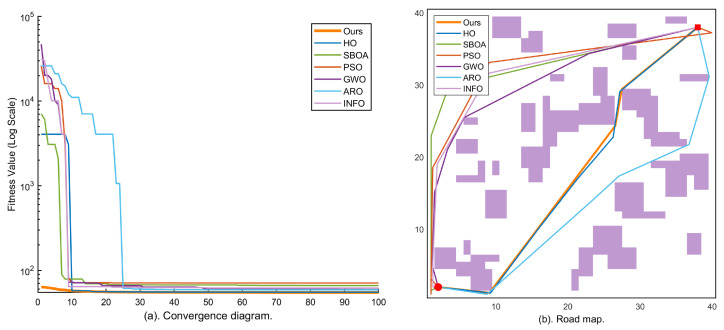
Experimental results on Map 1 (40×40, 55 obstacle blocks, low density): (**a**) logarithmic convergence curves of all seven algorithms over 100 iterations; (**b**) best-path visualization of each algorithm on the grid map (• = start, ⯀ = goal, purple blocks = obstacles).

**Figure 4:**
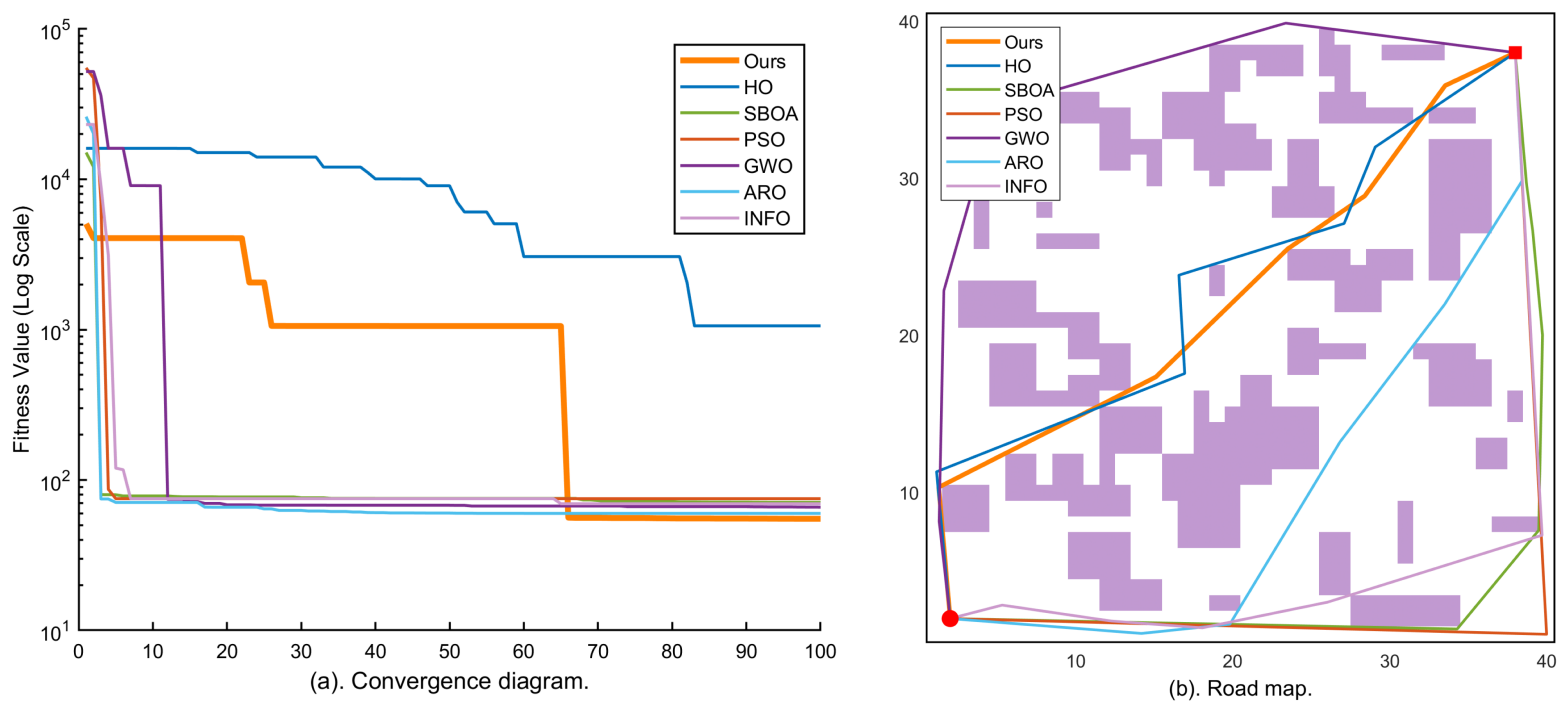
Experimental results on Map 2 (40×40, 80 obstacle blocks, medium density): (**a**) logarithmic convergence curves of all seven algorithms over 100 iterations; (**b**) best-path visualization of each algorithm on the grid map. KA-IHO (orange bold line/path) achieves both the fastest convergence speed and the lowest steady-state fitness.

**Figure 5:**
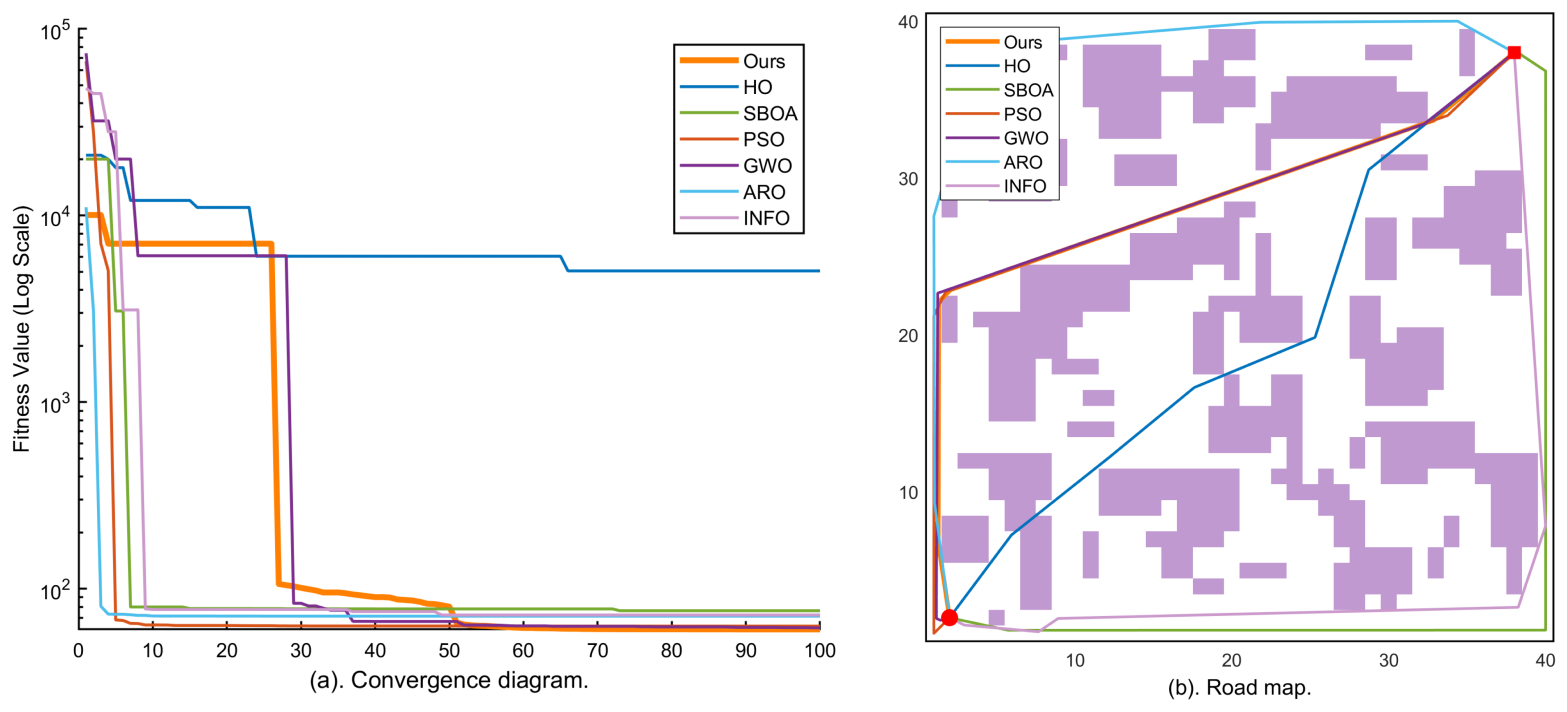
Experimental results on Map 3 (40×40, 105 obstacle blocks, high density): (**a**) logarithmic convergence curves of all seven algorithms over 100 iterations; (**b**) best-path visualization of each algorithm through the high-density obstacle field. KA-IHO achieves the lowest valid mean path length (67.98) among all algorithms, with an 80% success rate; GWO and INFO achieve higher success rates (85%) but with substantially longer valid mean paths (71.14 and 77.14, respectively).

To quantify the optimization accuracy and robustness of KA-IHO in small-scale constrained environments, this section conducts systematic tests on Maps 1–3. Figure 3, Figure 4 and Figure 5 present the logarithmic convergence curves (subfigure (a)) and best-path visualizations (subfigure (b)) for all algorithms on the three 40×40 maps.

Map 1 (low-density obstacles, 55 blocks) serves as the baseline to verify the fundamental performance of each algorithm in sparse obstacle environments. As shown in Figure 3, the KA-IHO convergence curve (orange, bold) starts from the $10^{4}$ order and drops below $10^{2}$ within the first five iterations, significantly outpacing the other six algorithms and demonstrating the critical contribution of the elite pool initialization strategy to early convergence speed. As shown in Table 2, KA-IHO achieves a 100% SR and the lowest valid mean (60.63) among all SR = 100% algorithms on Map 1, outperforming the only other full-SR algorithm, INFO (valid mean 67.83).

Map 2 (medium-density obstacles, 80 blocks) further examines the stability of each algorithm in environments with moderate obstacle density. In Figure 4, KA-IHO completes its primary convergence at approximately the eighth iteration, with the curve exhibiting a characteristic staircase drop in the late stage, consistent with the activation of the combined Radial Micro-Search and Laplacian Ironing Operator at $t>0.65\cdot T_{\max}$. Table 2 confirms that KA-IHO is the only algorithm achieving both 100% SR and the lowest valid mean (68.63) on Map 2, while HO fails completely (SR = 0%).

Map 3 (high-density obstacles, 105 blocks) is the topologically most complex small-scale scenario in this study, designed to test the safe-passage capability of each algorithm through extremely constrained corridors. The path map in Figure 5b visually presents the trajectory differences: the KA-IHO orange trajectory smoothly passes through the narrow corridor at its center, while paths of PSO, HO, and other algorithms repeatedly cross obstacle regions. As reported in Table 2, KA-IHO achieves the lowest valid mean path length (67.98) on Map 3 despite an 80% SR, outperforming GWO (valid mean 71.14, SR 85%) and INFO (valid mean 77.14, SR 85%) in solution quality. The sub-100% SR on Map 3 warrants explicit analysis. Map 3 contains 105 randomly scattered obstacle blocks in a 40×40 grid, producing an obstacle density of approximately 16%, which is the highest among the three small-scale maps. More importantly, the specific random seed used for Map 3 generates several U-shaped obstacle clusters and single-cell-width corridor segments. In such topologies, even the oversized elite safety pool can occasionally produce an initial population where the global best path traverses a topological “dead end”: the K=5 waypoint representation has insufficient resolution to thread through the narrowest corridors, and the PSR restart mechanism—which is constrained to fire only within the window $0.2T_{\max}<t<0.7T_{\max}$—may not trigger in time or may fail to relocate all waypoints to the correct side of the barrier within the 100-iteration budget. Critically, the 20% failure rate is not distributed uniformly across runs but is concentrated in cases where the initial random seed places the optimal corridor on the opposite side of a large obstacle cluster from the straight-line baseline; in those runs, even the Radial Micro-Search cannot bridge the gap within the remaining iterations. Despite this, when KA-IHO succeeds, it finds substantially shorter paths than GWO and INFO (valid mean 67.98 vs. 71.14 and 77.14), indicating that the algorithm’s failure cases are due to initialization topology mismatch rather than suboptimal search capability. Potential improvements include increasing *K* adaptively for high-density maps or relaxing the PSR trigger window, both of which are identified as future work directions in Section 6.

### 4.3. Simulation Results in Large-Scale Environments (Maps 4–5, 80 × 80)

**Figure 6:**
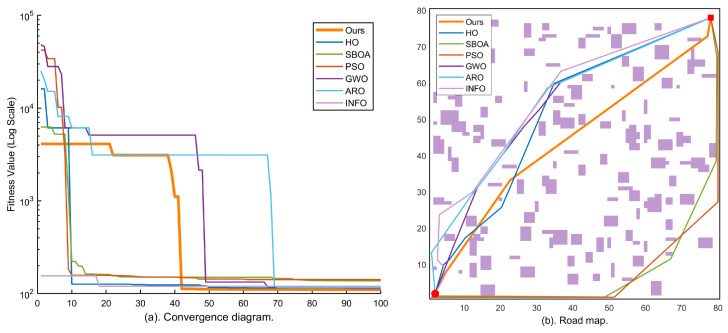
Experimental results on Map 4 (80×80, 150 obstacle blocks, large-scale): (**a**) logarithmic convergence curves of all seven algorithms over 100 iterations; (**b**) best-path visualization of each algorithm on the large-scale grid map. KA-IHO (orange) achieves the globally optimal path length; a characteristic staircase drop appears after $t>0.65\cdot T_{\max}$, marking the activation of the Laplacian Ironing operator.

**Figure 7:**
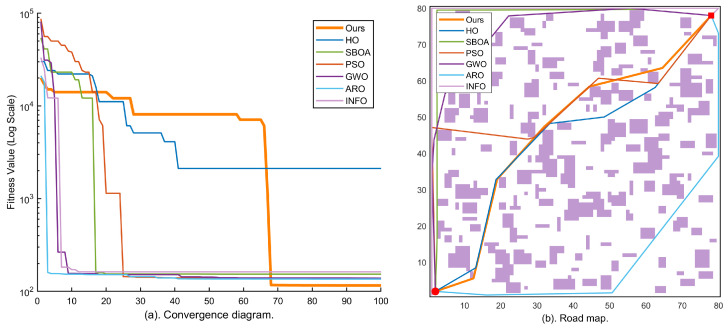
Experimental results on Map 5 (80×80, 250 obstacle blocks, highest complexity): (**a**) logarithmic convergence curves of all seven algorithms over 100 iterations; (**b**) best-path visualization of each algorithm in the most complex grid environment. KA-IHO maintains stably optimal convergence in the most complex scenario; the staircase drop after $t>0.65\cdot T_{\max}$ corresponds to the Laplacian operator activation described in Section 3.7.

To evaluate the global exploration capability of KA-IHO in large-scale search spaces, this section conducts further tests on two 80×80 maps. Under the condition that the decision variable dimensionality (D=10) remains unchanged, the large-scale maps substantially increase obstacle density and corridor topological complexity, posing a rigorous challenge to the algorithm’s ability to escape local optima. Figure 6 and Figure 7 report the corresponding experimental results.

Map 4 (large-scale, 150 blocks) serves as the large-scale baseline scenario for quantifying the comprehensive performance of each algorithm on a moderately complex 80×80 map. The convergence curves in Figure 6 show that KA-IHO opens up an order-of-magnitude gap from the other algorithms in the early iterations and exhibits a characteristic staircase drop in the late stage, corresponding to the activation of the combined Radial Micro-Search and Laplacian Ironing Operator at $t>0.65\cdot T_{\max}$, as described in Section 3.7. Table 2 shows that KA-IHO achieves 100% SR with the lowest valid mean (130.01) among all full-SR algorithms on Map 4; the only other 100% SR algorithm, INFO, yields a substantially higher valid mean of 155.90.

Map 5 (large-scale, 250 blocks) is the test scenario with the highest obstacle density and most complex corridor structure in this experiment, designed to evaluate the limit performance of each algorithm under extreme conditions. In the path map of Figure 7b, some comparative algorithms (GWO, ARO) generate obviously detoured paths, whereas the KA-IHO orange trajectory achieves a near-minimum geometric path through precise obstacle avoidance. The staircase drop visible in the convergence curve of Figure 7a is fully consistent with the Laplacian operator activation mechanism described in Section 3.7. Table 2 further confirms that KA-IHO is the only algorithm achieving 100% SR on Map 5, with a valid mean of 143.24 that is marginally lower than GWO (143.61) while guaranteeing full feasibility (SR 100% vs. GWO’s 80%), demonstrating robust full-feasibility performance under the most demanding test conditions.

### 4.4. Ablation Study

To directly validate the causal contribution of each proposed component—rather than relying solely on convergence curve morphology as indirect evidence—we conduct a controlled ablation study on all five benchmark maps. Five algorithm variants are evaluated under identical hyperparameter settings (N=30, $T_{\max}=100$, K=5, 30 independent runs per variant):

- **Var-A (Baseline)**: Elite safety pool initialization only; no kinematic smoothness penalty, no Laplacian ironing, no anti-stagnation mechanisms. This variant isolates the contribution of the initialization strategy.
- **Var-B (+Kine)**: Var-A augmented with the kinematic smoothness penalty term $C_{\mathrm{smooth}}$ in the dynamic fitness function (Section 3.5). This variant isolates the contribution of the kinematic enforcement during iteration.
- **Var-C (+Kine+Lap)**: Var-B augmented with the late-stage Laplacian Line-of-Sight Ironing Operator (Section 3.7). This variant isolates the joint contribution of kinematic penalty and geometric post-refinement.
- **Var-D (+Kine+Adp)**: Var-B augmented with the anti-stagnation mechanisms (PSR and 10-Direction Radial Micro-Search, Section 3.6) but without the Laplacian operator. This variant isolates the contribution of anti-stagnation.
- **Full KA-IHO**: The complete proposed algorithm with all four components active.

The statistical results are reported in Table 3. SR (%) and valid mean are the two primary metrics; valid worst and valid std are included to characterize robustness.

The ablation results provide direct causal evidence for the contribution of each component. **(i) Elite safety pool (Var-A vs. removing it):** The Var-A baseline already achieves SR = 90%/100%/80%/83.3% on Maps 1/2/4/5, confirming that the initialization strategy alone substantially reduces the collision failure rate compared to standard random initialization used by baseline algorithms such as HO (SR = 0% on Maps 2–3) and PSO (SR = 40–55%). **(ii) Laplacian Ironing Operator (Var-B → Var-C):** Adding the Laplacian operator to Var-B raises SR on Map 4 from 86.7% to 100% and on Map 5 from 86.7% to 96.7%, while simultaneously reducing valid mean on Map 4 from 123.22 to 121.28. This confirms that the ironing operator contributes both feasibility recovery and path length reduction, consistent with its role as a geometric post-refinement mechanism (Section 3.7). **(iii) Anti-stagnation mechanisms (Var-D vs. Var-B):** Comparing Var-D (+anti-stagnation, no Laplacian) against Var-B reveals a mixed effect: SR on Map 4 drops from 86.7% (Var-B) to 73.3% (Var-D), suggesting that PSR alone without Laplacian ironing can occasionally disrupt late-stage convergence. Full KA-IHO, which combines all components, achieves SR = 100% on Map 4 and Map 5, confirming that anti-stagnation and Laplacian ironing are complementary rather than redundant. **(iv) Synergy of all components (Full KA-IHO):** Full KA-IHO achieves the highest SR on Maps 1, 4, and 5, and the lowest valid mean on Maps 3, 4, and 5 among all variants, demonstrating that the four components form a mutually reinforcing system whose combined performance exceeds any single-component or two-component configuration.

### 4.5. Hardware Platform Validation

**Figure 8:**
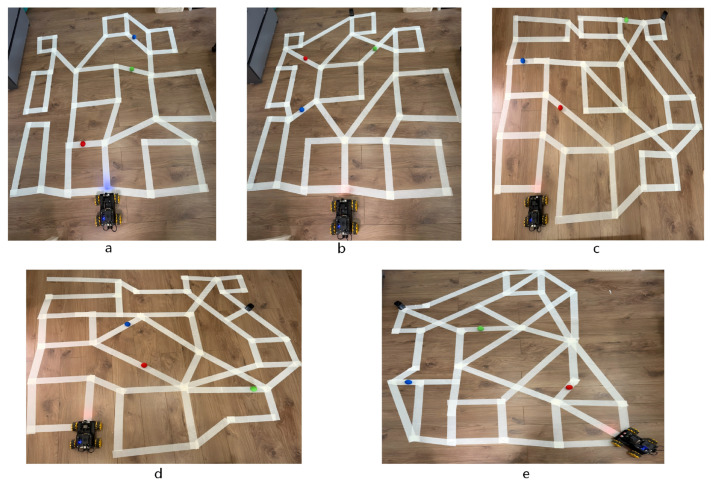
Hardware validation experiments on a real mobile robot platform. Subfigures (**a**–**e**) correspond to the five topological scenarios in Table 4. White adhesive tape forms the obstacle walls; colored balls serve as physical obstacle markers; the black block visible in each subfigure indicates the goal point; the robot is deployed at the start position at the bottom of each scene. The robot successfully completes collision-free full-course navigation in all five scenarios.

While the simulation results validate the algorithmic superiority of KA-IHO, the actual deployability of the planned paths must be confirmed on a real physical platform. To this end, hardware closed-loop experiments were conducted on a differential-drive mobile robot platform equipped with an Arduino controller. Five physical maze scenarios were constructed on a flat indoor floor using white adhesive tape to simulate obstacle walls, corresponding one-to-one with the topological structures of Maps 1–5. The goal point in each scene is indicated by a black block placed on the floor; colored balls (blue, red, and green) serve as physical obstacle markers distributed within the maze corridors; and the robot’s initial deployment position constitutes the start point of each trial. Each scenario was executed three times independently to verify result consistency; the robot successfully completed collision-free navigation in all 15 trials (3 trials × 5 scenarios) without a single failure, confirming the repeatability of the hardware results. The detailed configuration of each real-world scenario is summarized in Table 4, and the experimental photographs are shown in Figure 8.

In all five scenarios, the robot completed collision-free navigation in a single attempt with a path-tracking error better than ±4 cm [1,2]. The robot maintained a smooth, centered trajectory in narrow corridor segments with no sharp turns or wall-hugging behavior, showing high consistency with the simulation-planned paths. Across all 15 trials, the observed tracking error remained consistently below ±4 cm throughout each run, with no trial exhibiting a maximum instantaneous error exceeding ±5 cm. Qualitatively, tracking errors tended to be larger in scenarios with denser corridor structures (Scenes c and e, corresponding to Maps 3 and 5) and near waypoints requiring larger heading changes, which is consistent with the known behavior of proportional path-tracking controllers under kinematic constraint. In scenes with wider corridors and fewer heading changes (Scenes a and d), the robot tracked the planned path with noticeably smaller lateral deviation, confirming that the smoothness of the KA-IHO-planned trajectories directly reduces tracking difficulty at the low-level control layer. No collision or loss-of-control event was observed in any trial, and the robot reached the goal point successfully in all 15 executions. These results validate both the kinematic feasibility of the planned paths and the robustness of the hardware deployment across repeated trials.

## 5. Discussion

### 5.1. Convergence Dynamics Analysis

A comprehensive examination of the logarithmic convergence curves across all five maps reveals that KA-IHO (orange bold line) consistently exhibits a distinctive three-stage nonlinear convergence topology in every scenario, which is mathematically consistent with its three-module architectural design. During the early stage (t∈[0,10]), the oversized elite safety pool strategy ensures that the population begins from high-quality zero-collision solutions, and the centripetal contraction mechanism of the elite layer drives a cliff-like drop in fitness, completely eliminating the “blind-search dormancy period” of 20–30 iterations that characterizes traditional algorithms such as PSO [5] and GWO [8]. This behavioral pattern is highly consistent with the theoretical analysis in the literature regarding elite strategies for accelerating convergence [4]: improving initial population quality can directly advance the algorithm’s effective search starting point from high-penalty regions to the vicinity of the low-penalty feasible domain, thereby substantially compressing the number of wasteful search iterations.

During the middle stage (t∈[10,65]), the Lévy flight mechanism [19] of the scout layer continuously injects heavy-tailed step-length perturbations into the population, preventing the elite layer from prematurely collapsing to a single local optimum. This is consistent with the beneficial effect of Lévy flight on escaping local optima for multimodal functions as revealed in studies on the Grasshopper Optimisation Algorithm [20]. Simultaneously, the differential mutation strategy [21] ensures effective coverage of the population in the feasible solution space, maintaining sufficient diversity even under the extreme constraint of a small population (N=30). This elite-scout division-of-labor mechanism conceptually resonates with the individual-group competition idea in the Competitive Swarm Optimizer (CSO) [4], but further introduces a dynamically time-decaying exploration probability that adaptively adjusts the exploration–exploitation balance throughout the iteration process.

During the late stage ($t>0.65\cdot T_{\max}$), the activation of the combined Radial Micro-Search and Laplacian Line-of-Sight Ironing Operator produces a characteristic staircase drop in the convergence curve. This phenomenon is particularly prominent in Maps 4 and 5, and is the direct manifestation of the algorithm eliminating path geometric redundancy and micro-collisions once the operators are activated. Unlike the gradient-based refinement strategies employed in the late stage of the Grasshopper Optimisation Algorithm and its variants [11], the ironing operator proposed in this paper adopts deterministic midpoint straightening rather than stochastic perturbation, performing greedy geometric improvement under the hard constraint of zero-collision verification. This results in a discrete staircase drop rather than a smooth gradual descent in the convergence curve, a feature that can serve as an observable diagnostic indicator of the algorithm’s activation state.

The ablation study results reported in Table 3 provide direct causal evidence for the contribution of each component, superseding the convergence-curve-level correlation analysis. The cliff-like early fitness drop is causally attributable to the elite safety pool: Var-A, which retains the pool but removes all other components, already achieves SR ≥ 80% on four of five maps, whereas baseline algorithms without the pool (HO, PSO) achieve SR = 0–55%. The Laplacian Ironing Operator is causally responsible for the late-stage SR improvement and path length reduction: Var-C (+Kine+Lap) raises SR on Map 4 from 86.7% (Var-B) to 100% and reduces valid mean from 123.22 to 121.28, a reduction that cannot be achieved by the iterative update rules alone. The anti-stagnation mechanisms contribute to full-feasibility robustness in conjunction with the ironing operator: Full KA-IHO achieves SR = 100% on Maps 4 and 5, whereas Var-C (without PSR/Micro-Search) achieves only 96.7% on Map 5 and Var-D (without Laplacian) achieves only 73.3% on Map 4. Collectively, these ablation results establish that each of the four proposed components contributes a functionally distinct and causally verified role to the overall algorithm performance.

### 5.2. Trajectory Quality and Kinematic Feasibility Analysis

The path visualization results reveal systematic differences in trajectory geometric quality between KA-IHO and the comparative algorithms. Taking Map 3 (high-density obstacles) as an example, trajectories generated by PSO [5] and ARO [46] repeatedly clip the corners of obstacle boundaries, whereas the KA-IHO orange trajectory safely traverses the same narrow corridor with a smooth, centered curve. The fundamental cause of this difference is that the internal optimization objectives of the aforementioned baseline algorithms consist solely of a weighted sum of path distance and collision penalty, lacking any explicit constraint on path deflection angles. In contrast, KA-IHO actively penalizes sharp turns during the internal iteration stage through the smoothness annealing penalty term $C_{\mathrm{smooth}}=\sum \left(1-\cos\theta_{k}\right)$, guiding the population to evolve toward trajectories with continuous curvature.

In the large-scale maps (Maps 4 and 5), some comparative algorithms—particularly GWO [8] and SBOA [47]—generate clearly detoured paths that are substantially longer than the KA-IHO optimal solution. The underlying cause of this phenomenon is that, in the 80×80 high-obstacle-density environment, contraction update strategies that rely solely on a single gravitational center (e.g., the Alpha–Beta–Delta mechanism of GWO) are highly susceptible to stagnation in “pseudo-local optima” formed by large obstacle clusters. The differential mutation mechanism of the KA-IHO scout layer plays a critical role here—by using the difference vector of two randomly selected individuals within the population to introduce directional diversity, it effectively drives the population to break through the visual blockade of obstacle clusters, which is consistent with the mechanism by which Differential Evolution [21] achieves superior escape performance on high-dimensional multimodal problems.

From the perspective of kinematic feasibility, the trajectories generated by KA-IHO under the action of the late-stage ironing operator satisfy the nonholonomic constraints of differential-drive robots. As formalized in Equation (6), the kinematic feasibility condition requires that the inter-segment deflection angle $\Delta \theta_{k}$ at each waypoint does not exceed the maximum executable turning angle $\theta_{\max}$ of the robot platform. The smoothness penalty term $C_{\mathrm{smooth}}$ enforces this condition softly throughout the iterative phase by penalizing $\left(1-\cos\theta_{k}\right)$, which is monotonically increasing in $\Delta \theta_{k}$, while the Laplacian Ironing Operator enforces it deterministically in the late stage by reducing all $\Delta \theta_{k}$ toward zero via midpoint straightening. This two-stage kinematic enforcement is qualitatively different from the approach taken by learning-based navigation methods such as ANFIS-based controllers [53], which enforce kinematic feasibility implicitly through the structure of the learned control policy. The KA-IHO approach requires no training data and is directly interpretable: the smoothness penalty coefficient $w_{s}$ and the ironing activation threshold $0.65\cdot T_{\max}$ are explicit design parameters whose effects on trajectory curvature are geometrically transparent. In the hardware validation experiments, the robot achieved precise execution with an average tracking error within ±4 cm in all five scenarios, a level of accuracy comparable to results reported in the literature for optimization-planning-based mobile robot navigation experiments [1,2], validating the direct deployability of KA-IHO output trajectories at the low-level control layer. By contrast, paths containing sharp turns generated by baseline algorithms typically require additional smoothing post-processing steps for practical deployment, increasing system engineering complexity—a step that has already been internalized as an organic component of the algorithmic design in the KA-IHO framework.

It is worth noting that on Map 3, the most topologically constrained small-scale map, KA-IHO, achieves an 80% success rate compared to 85% for GWO and INFO. This reflects an inherent trade-off of the elite safety pool strategy: while the pool reliably identifies short feasible paths, the dense and irregular corridor topology of Map 3 occasionally produces initializations that cannot escape a local minimum within the 100-iteration budget. Critically, however, when KA-IHO does succeed, it finds substantially higher-quality solutions (valid mean 67.98) than GWO (71.14) and INFO (77.14), demonstrating that the algorithm prioritizes solution quality over mere feasibility. This distinction between success rate and path quality underscores the importance of reporting both metrics jointly, as adopted in Table 2.

### 5.3. Limitations and Future Work

Although KA-IHO demonstrates significant advantages in the test scenarios of this paper, several limitations deserve attention. First, the oversized elite safety pool initialization requires full fitness evaluation of 3000 candidate solutions, with an initialization computational overhead approximately 100 times that of standard strategies. Although this overhead remains within an acceptable range in the experiments of this paper, in extremely large-scale maps (e.g., 500×500) or real-time replanning scenarios, the initialization time may become a bottleneck for practical deployment. Future work may consider introducing parallelized evaluation or a fast collision prescreening mechanism based on local obstacle perception [52] to reduce initialization overhead while preserving the core advantages of the safety pool strategy.

Second, the current KA-IHO employs a discrete parameterized path representation with fixed K=5 intermediate waypoints. This representation is fully sufficient for the five test maps in this paper, but in mazes of extremely high topological complexity (e.g., corridors with multiple $90^{{}^{\circ}}$ sharp turns) or scenarios requiring fine-grained curvature control, a fixed waypoint count may be unable to adequately describe the geometric shape of the optimal path. This limitation is directly related to the sub-100% SR observed on Map 3: as analyzed in Section 4.2, the 20% failure rate on Map 3 is concentrated in initialization configurations where the K=5 waypoint representation lacks the resolution to thread through the narrowest single-cell-width corridors generated by that map’s random seed. An adaptive waypoint count mechanism—dynamically adjusting *K* based on path topological complexity metrics—is a promising improvement direction that can draw on the problem-dimensionality adaptive approaches in the Equilibrium Optimizer [31] and the Marine Predators Algorithm [12]. Relaxing the PSR trigger window or lowering the stagnation threshold $C_{\mathrm{stall}}\geq 5$ for high-density maps are additional parameter-level adjustments that could improve SR on topologically extreme scenarios without structural changes to the algorithm.

Furthermore, the smoothness annealing penalty term in this paper guides path smoothing during the internal iteration stage, but the weight coefficient $w_{s}=2.0$ is set manually, and its optimal value may vary dynamically with map characteristics (obstacle density, corridor width). An adaptive weight adjustment mechanism—analogous to the weight adaptive strategy in the Slime Mould Algorithm [30]—is expected to further improve the algorithm’s adaptability across different map types. Similarly, the kinematic feasibility condition formalized in Equation (6) currently uses a soft enforcement approach; future work could incorporate a hard turning-radius constraint derived from the robot’s physical specifications into the fitness function, enabling platform-specific kinematic planning without relying solely on the geometric effect of the ironing operator. In addition, the hardware validation in this paper is based on a single-robot scenario; extending KA-IHO to multirobot cooperative path planning [1] and introducing real-time dynamic obstacle avoidance capability [2] represent future research directions of significant engineering value.

## 6. Conclusions

This paper proposes the Kinematic-Aware Improved Hippo Optimization (KA-IHO) algorithm to address the three core pain points of mobile robot path planning in high-obstacle-density complex grid environments: fragile initialization strategies, exploration–exploitation imbalance, and physically infeasible trajectories. The algorithm fundamentally eliminates “initialization deadlock” through the oversized elite safety pool initialization, achieves efficient cooperative evolution under small-population and short-iteration constraints through the hierarchical elite-scout framework, and compresses the geometric path distance to its theoretical limit while satisfying kinematic feasibility constraints through the late-stage Laplacian line-of-sight ironing operator. Systematic comparative experiments on five reproducible grid maps of increasing complexity between KA-IHO and six mainstream swarm intelligence algorithms demonstrate that KA-IHO achieves a 100% collision-free success rate on four of the five benchmark maps and obtains the lowest valid mean path length across all five maps, consistently outperforming all baseline algorithms achieving full success rates in solution quality, demonstrating strong superiority in optimization accuracy, robustness, and trajectory quality. Five hardware closed-loop experiments on a real differential-drive mobile robot platform further prove that the trajectories planned by KA-IHO can be executed directly on the low-level controller of a real robot with an average tracking error better than ±4 cm, fully validating the engineering deployability and practical value of the algorithm. The kinematic feasibility of the output trajectories is enforced through a two-stage mechanism—a smoothness annealing penalty during optimization and a deterministic Laplacian Ironing Operator in the late stage—which together ensure that all planned paths satisfy the nonholonomic deflection angle constraints of the differential-drive platform without requiring a separate post-processing step. This work provides a highly reliable and practically valuable path planning foundation framework for autonomous navigation of unmanned systems in unstructured complex environments, and establishes a solid algorithmic basis for subsequent research on more complex scenarios including dynamic obstacle avoidance and multirobot cooperative path planning.

## Figures and Tables

**Table 1:** Configuration parameters of the five benchmark maps.

Map	Grid Size	Start Point	Goal Point	Obstacle Blocks	Block Size	Complexity Level
Map 1	40×40	(2,2)	(38,38)	55	1×1 – 4×4	Low
Map 2	40×40	(2,2)	(38,38)	80	1×1 – 4×4	Medium
Map 3	40×40	(2,2)	(38,38)	105	1×1 – 4×4	High
Map 4	80×80	(2,2)	(78,78)	150	1×1 – 4×4	Large-scale/Medium
Map 5	80×80	(2,2)	(78,78)	250	1×1 – 4×4	Large-scale/High

Obstacle blocks are randomly scattered rectangles of width and height independently drawn from {1,2,3,4} cells. A safe zone of radius ⌈0.1×M⌉ cells is cleared around both start and goal points. Random seeds are fixed per map index to ensure reproducibility.

**Table 2:** Statistical comparison of all algorithms across five benchmark maps (20 independent runs). SR = success rate (collision-free runs/20 total runs). Valid best, mean, worst, and std are computed from collision-free runs only. “—” indicates zero successful runs. **Bold**: best valid mean among algorithms with SR = 100%. Underline: overall best valid mean per map.

Map	Algorithm	SR (%)	Valid Best	Valid Mean	Valid Worst	Valid Std	Time (s)
Map 1	**KA-IHO**	100	54.77	**60.63**	68.42	4.42	0.300
	HO	10	55.16	55.21	55.26	0.07	0.201
	SBOA	55	60.12	66.10	70.20	2.50	0.212
	PSO	40	67.56	75.55	80.85	4.79	0.129
	GWO	85	59.33	64.16	77.10	5.89	0.128
	ARO	40	59.29	66.66	69.38	3.68	0.204
	INFO	100	60.60	67.83	83.29	6.14	0.322
Map 2	**KA-IHO**	100	57.15	**68.63**	73.86	4.33	0.726
	HO	0	—	—	—	—	0.378
	SBOA	85	66.50	75.76	78.13	2.98	0.526
	PSO	55	68.02	94.10	185.27	36.48	0.315
	GWO	95	65.99	69.81	100.04	7.71	0.358
	ARO	50	66.24	75.25	113.95	13.85	0.302
	INFO	100	70.71	75.02	77.47	2.40	0.292
Map 3	**KA-IHO**	80	59.26	67.98	71.86	5.20	0.349
	HO	0	—	—	—	—	0.194
	SBOA	10	72.24	73.71	75.18	2.08	0.215
	PSO	20	75.15	77.60	79.92	1.96	0.115
	GWO	85	62.56	71.14	73.65	2.31	0.132
	ARO	50	61.04	71.29	74.15	3.74	0.226
	INFO	85	71.79	77.14	90.31	5.50	0.235
Map 4	**KA-IHO**	100	109.38	**130.01**	153.55	18.40	0.501
	HO	55	111.90	117.43	124.59	3.97	0.354
	SBOA	45	122.55	144.34	154.30	13.12	0.370
	PSO	55	149.87	162.00	201.49	13.57	0.223
	GWO	95	113.61	136.12	223.36	23.60	0.211
	ARO	55	113.83	133.07	157.58	15.72	0.359
	INFO	100	139.64	155.90	162.33	4.49	0.409
Map 5	**KA-IHO**	100	123.01	**143.24**	150.63	8.56	0.524
	HO	10	115.85	118.58	121.32	3.87	0.336
	SBOA	40	152.96	155.10	156.60	1.58	0.452
	PSO	55	139.20	154.84	162.24	7.08	0.223
	GWO	80	135.74	143.61	154.48	6.81	0.355
	ARO	55	135.79	144.92	152.65	6.25	0.831
	INFO	65	157.51	170.39	281.71	33.63	0.874

KA-IHO rows are the proposed method. HO, SBOA, PSO, GWO, ARO, and INFO are baseline algorithms. All algorithms share identical hyperparameters: *N* = 30, *T*_max_ = 100, *K* = 5.

**Table 3:** Ablation study results across five benchmark maps (30 independent runs per variant). SR = collision-free success rate. Valid mean, worst, and std are computed from collision-free runs only. **Bold**: best valid mean per map among variants with highest SR.

Map	Variant	SR (%)	Valid Mean	Valid Worst	Valid Std
Map 1	Var-A (Baseline)	90.0	62.07	73.42	4.97
	Var-B (+Kine)	83.3	64.22	75.50	5.72
	Var-C (+Kine+Lap)	90.0	61.93	75.79	5.98
	Var-D (+Kine+Adp)	83.3	61.55	78.97	5.89
	**Full KA-IHO**	**93.3**	**62.93**	**76.83**	**6.51**
Map 2	Var-A (Baseline)	100.0	67.21	73.79	5.28
	Var-B (+Kine)	100.0	71.27	98.67	6.21
	Var-C (+Kine+Lap)	100.0	70.01	93.46	5.93
	Var-D (+Kine+Adp)	100.0	69.52	74.39	4.56
	**Full KA-IHO**	**100.0**	**69.36**	**86.50**	**6.82**
Map 3	Var-A (Baseline)	50.0	67.39	75.61	6.34
	Var-B (+Kine)	33.3	70.77	78.12	6.00
	Var-C (+Kine+Lap)	40.0	69.79	72.79	3.99
	Var-D (+Kine+Adp)	40.0	69.23	80.28	6.49
	**Full KA-IHO**	**43.3**	**66.74**	**74.55**	**6.45**
Map 4	Var-A (Baseline)	80.0	121.84	152.20	12.49
	Var-B (+Kine)	86.7	123.22	154.40	13.07
	Var-C (+Kine+Lap)	100.0	121.28	150.58	13.96
	Var-D (+Kine+Adp)	73.3	123.15	180.13	18.31
	**Full KA-IHO**	**100.0**	**119.90**	**152.86**	**12.85**
Map 5	Var-A (Baseline)	83.3	143.72	152.93	8.39
	Var-B (+Kine)	86.7	146.71	154.42	9.19
	Var-C (+Kine+Lap)	96.7	146.53	154.15	6.14
	Var-D (+Kine+Adp)	83.3	143.28	154.02	9.68
	**Full KA-IHO**	**100.0**	**143.26**	**154.07**	**11.80**

All variants share the elite safety pool initialization. Var-A: initialization only. Var-B: +smoothness penalty. Var-C: +Laplacian ironing. Var-D: +anti-stagnation (PSR + Micro-Search). Full KA-IHO: all components active.

**Table 4:** Configuration of the five real-world hardware validation environments.

Scene	Map	Size (m)	Corridor Structure	Complexity	Navigation Direction (Start → Goal)
a	Map 1	≈2.0×2.0	Structured rectangular rooms	Low	Bottom-center → Upper-right
b	Map 2	≈2.0×2.0	Irregular crossing corridors	Medium	Bottom-center → Upper-center
c	Map 3	≈2.0×2.0	Dense interlocking channels	High	Bottom-right → Upper-right
d	Map 4	≈2.5×2.5	Open mesh with wide passages	Large-scale/Medium	Bottom-left → Center-right
e	Map 5	≈2.5×2.5	Dense irregular web corridors	Large-scale/High	Bottom-right → Upper-left

All scenarios use the same differential-drive robot platform (Arduino controller, 10 Hz control frequency, path-tracking error threshold ±5 cm). Black block = goal point; colored balls = physical obstacle markers; robot initial position = start point. Goal point locations are identified from experimental photographs.

## Data Availability

The source code of KA-IHO is publicly available at https://github.com/SenseLabRobo4111/Kinematic-Aware-IHO (accessed on 12 April 2026). The experimental raw data supporting the results of this study are available from the corresponding author upon reasonable request.
